# The giant deep-sea octopus *Haliphron atlanticus* forages on gelatinous fauna

**DOI:** 10.1038/srep44952

**Published:** 2017-03-27

**Authors:** H.J.T. Hoving, S.H.D. Haddock

**Affiliations:** 1 GEOMAR, Helmholtz Centre for Ocean Research Kiel, Düsternbrooker Weg 20, 24105 Kiel, Germany; 2 Monterey Bay Aquarium Research Institute, CA 95039, Moss Landing, USA

## Abstract

Feeding strategies and predator-prey interactions of many deep-sea pelagic organisms are still unknown. This is also true for pelagic cephalopods, some of which are very abundant in oceanic ecosystems and which are known for their elaborate behaviors and central role in many foodwebs. We report on the first observations of the giant deep-sea octopus *Haliphron atlanticus* with prey. Using remotely operated vehicles, we saw these giant octopods holding medusae in their arms. One of the medusae could be identified as *Phacellophora camtschatica* (the egg-yolk jelly). Stomach content analysis confirmed predation on cnidarians and gelatinous organisms. The relationship between medusae and *H. atlanticus* is discussed, also in comparison with other species of the Argonautoidea, all of which have close relationships with gelatinous zooplankton.

The pelagic ocean is the largest living space on the planet, and one that holds enormous biodiversity and biomass^1^. While general trophic relations between oceanic pelagic organisms can be reconstructed using various dietary tracers^2^, knowledge of prey choice and feeding behavior remains virtually unknown for many pelagic fauna. With the advancement of underwater technology, deep-sea *in situ* observations are revealing novel behaviors of deep-sea organisms. Examples include luring in deep-sea siphonophores^3^ and squids^4^,^5^, carnivory and prey specialization in ctenophores^6^ and medusae^7^, and detritivory in cephalopods^8^.

Cephalopods are a group of molluscs that inhabit the marine environment from shallow reefs to the deep sea. Although their feeding behavior is commonly characterized as opportunistic, active hunting, and strictly carnivorous^9^, recent insights show a more diverse array of feeding strategies^10^. Cephalopods are among the largest inhabitants of the open ocean and play a pivotal role as prey of many large oceanic predators such as sperm whales, sharks, swordfish, tuna, and squid themselves^11^,^12^. One of these giant cephalopods is *Haliphron atlanticus* (Alloposidae), a pelagic species that may reach up to 4 meters in total length and up to 75 kg^13^ and is consumed by sperm whales, blue sharks, and pelagic fishes^14^,^15^

*Haliphron atlanticus* has a wide geographical distribution, and while it occurs in mesopelagic and bathypelagic waters of the open ocean, it is often associated with continental slopes, both in the pelagic water column as well as close to the bottom^16^. This species exhibits one of the strongest examples of sexual size dimorphism among cephalopods, with the small males dwarfed by the females. The hectocotylized arm is enclosed in a sac close to the right eye of the male. Therefore it appears to have only seven arms, giving it the common name of the seven-arm octopus. Males mate with the female by completely releasing their hectocotylus with the spermatophore and attaching it to the female. After spawning, the female broods her eggs, which are attached to the oral surface of the arm bases^17^. It is unknown what kind of feeding strategy fuels the likely single reproductive cycle (semelparity). Knowledge of the diet of the species is restricted to examination of stomachs of two trawl-captured specimens. The contents included ‘non-cuticular red-coloured prey, most likely cnidarian (a coronate medusa like *Atolla* sp.)’ and small amphipods^13^, and the shrimp *Pandalus borealis*^18^. We report *in-situ* observations of *Haliphron atlanticus* encountered during deep-sea dives with a remotely operated vehicle, as well as stomach content analysis of museum specimens.

## Results

**Figure 1:**
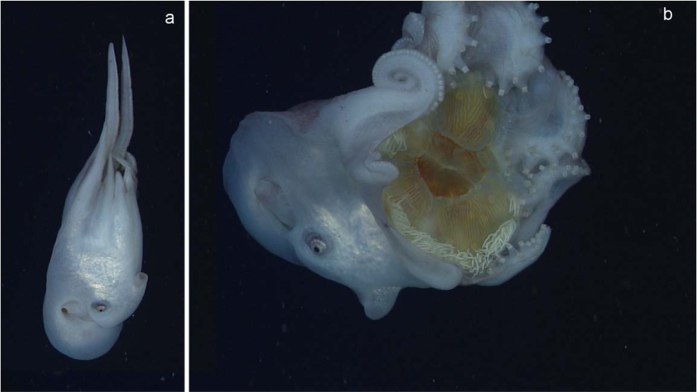
*Haliphron atlanticus* observed by the remotely operated vehicle *Doc Ricketts* in the Monterey Submarine Canyon at 378 m of depth. (**a**) The individual is swimming down. (**b**) The individual with the *Phacellophora* specimen visible in the arm crown. Estimated mantle width (vertically distance in panel b) is 29 cm.

Three *Haliphron* were observed by MBARI’s remotely operated vehicles, two in the Monterey Submarine Canyon (at 390 and 520 m), and one off Hawaii (250 m). These observations included a small individual which was observed with gelatinous material in its arms. It was impossible to identify the organism that was being held. Another individual also had a yellow mass in its arms, but since the arms were never opened, the nature of the mass remained concealed. The large size of the specimen suggested it was a brooding female^16^, although our current observation may alter this interpretation. Both specimens are shown in Young^16^. Our third observation, from July 2013, involved a large female *H. atlanticus* (mantle width ~29 cm; Fig. 1). This specimen held the medusa *Phacellophora camtschatica* (egg-yolk jelly) which had lost all of its stomach and oral arms, and most of its tentacles. The observation of this *H. atlanticus* from the ROV (Fig. 1b) showed that the outer surface of the medusa bell was held by the arms and suckers. The animal was able to swim while holding the medusa, and when the arms were closed, the medusa was not visible. The *Haliphron* appeared to have bitten through the bell from the outside, as the beak was sometimes visible in the center of the subumbrella of the medusa (Fig. 1b).

*Haliphron atlanticus* specimens (n = 5) accessioned in the Hamburg Zoological Museum (Table S1) were examined and all specimens had body parts of gelatinous fauna in their crop and stomachs (Supplementary Figure S1). Two of the specimens had tentacles and lightly pigmented frilled material (one with fine tentacles), consistent with scyphozoan oral arms (Fig S1a), as we would expect from our *in situ* observation. Another specimen had brick red pigmentation and tentacles of a coronate scyphozoan (Fig S1b), similar to the previous report of a coronate medusa in gut contents^13^. One stomach had the remains of the stem and seven intact tentilla of a physonect siphonophore (Fig S1c), likely in the family Agalmatidae. The fifth specimen included the remains of a salp chain along with the only crustacean found in the stomach contents (Fig S1d). This crustacean was identified as an amphipod of the genus *Vibilia*, which is known to specialize in parasitizing salps^19^,^20^. This record corresponds with the amphipod gut-contents previously reported^13^, which may be indicative of ingestion as “by-catch” when the gelatinous host of the amphipods is consumed.

## Discussion

The only other information that existed previous to our observation was the finding of gelatinous fragments (identified to belong to a mesopelagic coronate medusa) and crustaceans in the crop of trawl captured *H. atlanticus*^13^,^18^. Our observations of two *Haliphron* holding on to a jellyfish combined with the finding of jellyfish fragments in the stomachs, in the literature and as observed here, strongly suggest that *H. atlanticus* feeds on gelatinous zooplankton. Other genera in the Argonautoidea are known to maintain symbiotic associations with gelatinous organisms, such as living in the barrels of salps or holding the tentacles of siphonophores. Such an association could be occurring with *Haliphron* as well, given that the clearest images show it holding the bell of the medusa with the fringe of tentacles intact, and the octopus’ beak protruding through a slit in the center of the bell. Fully establishing that there is a feeding relationship beyond the behavioral association requires more than just witnessing the association, and we believe that the combined evidence supports that the octopus feeds upon the medusa, at least initially in the relationship: Our five specimens and the published record of gelatinous remains in the stomachs of net-caught *Haliphron* specimens^13^ complements our *in-situ* observation that the oral arms and stomach of the medusa were missing from within the bell. This is similar to how fish^21^ and sea turtles^22^ feed on medusae, by initially targeting their more energy-dense oral arms and gonads.

*In situ* observations of *Haliphron* obtained previously repeatedly show that a mass is being held (and concealed) in between the arms^15^ (https://youtu.be/sw8zl5vrAu8? t=1m50s; Accessed 01/30/2017). Since the species is known to hold on to the eggs after spawning^17^, these masses may be eggs. Taking into account our current observations though, these concealed masses may also be the carcasses of gelatinous zooplankton.

There are other reports of cephalopods with gelatinous fauna in their stomachs^23^. Cnidaria, mostly *Velella*, accounted for more than 20% of the prey weight in market squid *Doryteuthis opalescens*^24^,^25^. The large onychoteuthid *Onykia robusta* had one *Velella* specimen in its jaws^26^. Vampire squid were reported with gelatinous material in their stomachs^7^. *Sthenoteuthis pteropus* consumed high numbers of *Pyrosoma atlantica* in the eastern tropical Atlantic^27^. Since these cephalopods (except vampire squid) are known to primarily consume crustacean and fish prey, feeding on gelatinous zooplankton may be incidental and part of their opportunistic feeding behavior. The observation of gelatinous prey ingestion by *Haliphron* adds to the variety of feeding strategies that can be found within the Cephalopoda.

The finding of *H. atlanticus* consuming medusae fits in the habits of argonautoid cephalopods, which apparently all have a relationship with gelatinous zooplankton at some point in their life. As juveniles, *Ocythoe tuberculata* are sometimes found inside the tests of large salps (e.g. *Thetys vagina*)^28^ (J. Milisen, pers. obs.). Juveniles of *Tremoctopus gracilis* carry tentacles of cnidarians, often Portuguese man of war, which are held by the suckers on their dorsal arms and may function as defense or for capturing prey^29^. *Argonauta argo* Linnaeus, 1758 is regularly seen to attach to jellyfish^16^,^30^, and a detailed examination was performed of an *Argonauta* that was observed clasping and attacking a *Phyllorhiza* scyphomedusa^31^. Heeger *et al*.^31^ wrote: “The formation of channels within the mesogloea of the jellyfish is of special importance. We conclude that the argonaut produced them by consuming parts of the exumbrella and mesogloea to connect to the gastral cavity of the medusa. This would enable the cephalopod to consume zooplankton from the gastrovascular system of the jellyfish via these channels while adhering to the exumbrella. Thereby the argonaut would use the efficient secondary mouth papillae of the oral arms of the jellyfish to collect prey items for itself.” The manner in which *Haliphron* holds the medusa in our observations is very similar to the description by Heeger *et al*.^31^. In *Haliphron* the medusa was held by the external bell with the oral part open within the folds of the arms, and the octopus’s beak sometimes protruding into the subumbrellar space. Therefore, in addition to feeding directly on jellyfishes, *Haliphron* may target the stomach contents of the medusa, or even use the medusa as a tool to obtain more nutritious prey that are captured by the fringe of tentacles clasped within the octopus arms. The daytime distribution of *Phacellophora* in Monterey Bay is shallower than the few observations of *Haliphron atlanticus* (53.4 meters; n = 137). Whether *Haliphron* swims up at night to capture these medusae or eats them when they are dead and sinking down, remains speculation.

*In situ* deep-sea observations have provided new insights in the importance of gelatinous fauna in the ocean and its foodwebs^5^,^32^. An increasing number of large oceanic organisms (e.g. tuna) are found to feed on gelatinous zooplankton^33^. The importance of gelatinous zooplankton in diet studies may traditionally have been underestimated since digestion rates of gelatinous zooplankton are higher than squid and fish prey, and no hard parts are left to track ingestion^34^. The importance of gelatinous fauna in diets was long questioned because the caloric values of gelatinous tissue were thought to be too low. *Haliphron* has very low mass-specific metabolic rates, comparable to some medusae^35^, and to maintain its routine metabolism, this cephalopod does not require a lot of energy. Therefore the ingested gelatinous tissue may be sufficient to support its energetic requirements. *Haliphron* may also eat only the nutritious parts of the jellyfish, *e.g*. stomach contents or gonads. Selective feeding on the nutritious gonads of various large deepwater cnidarians has been observed in ocean sunfish^36^. If *Haliphron* indeed has a large jellyfish component in its ingested prey, it coincides with the observations that some of the largest oceanic predators (ocean sunfish, leatherback turtles), can attain very large sizes on a gelatinous diet.

In the North Atlantic *H. atlanticus* is an important prey of sperm whales, swordfish and blue sharks^33^,^34^. The observation of *Haliphron* feeding upon *Phacellophora* and other gelatinous organisms indicate that *H. atlanticus* may be a trophic link between gelatinous zooplankton and top predators. This further confirms the pivotal role that cephalopods and gelatinous zooplankton constitute in oceanic foodwebs, channeling energy from the bottom to the top of the oceanic food chain.

## Additional Information

**How to cite this article:** Hoving, H.J.T. and Haddock, S.H.D. The giant deep-sea octopus *Haliphron atlanticus* forages on gelatinous fauna. *Sci. Rep.* **7**, 44952; doi: 10.1038/srep44952 (2017).

**Publisher's note:** Springer Nature remains neutral with regard to jurisdictional claims in published maps and institutional affiliations.

## Supplementary Material

Supplementary Information
