# Supplementary material for: The giant deep-sea octopus Haliphron atlanticus forages on gelatinous fauna
Source: Sci Rep. 2017 Mar 27;7:44952. doi: 10.1038/srep44952 (PMC5366804; doi:10.1038/srep44952)
Supplement: Supplementary Information [file srep44952-s1.pdf]

Supplementary information for Hoving and Haddock. "The giant deep-sea octopus *Haliphron atlanticus* forages on gelatinous fauna".

Table S1. Samples examined from the collections of the Hamburg Zoological Museum, with collection location and depth information where available. Four of the specimens are depicted in Figure S1.

Plate	CatNo	Area	Date	Latitude	Longitude	Depth m	Contents
-	12771	North Sea	May 1999	-	-	100	Medusa fragments
a	12659	Pacific Ocean	May 1975	30°40'N	116°19'W	-	Medusa + tentacles
b	12769	Pacific Ocean	Apr. 1975	28°40'N	119°05'W	300	Coronate medusa
c	73849	Canary Islands	Apr. 1971	17°22' N	022°58'W	600	Siphonophore
d	12646	Pacific Ocean	Apr. 1975	29°20'N	121°08'W	400	Salp + Amphipod

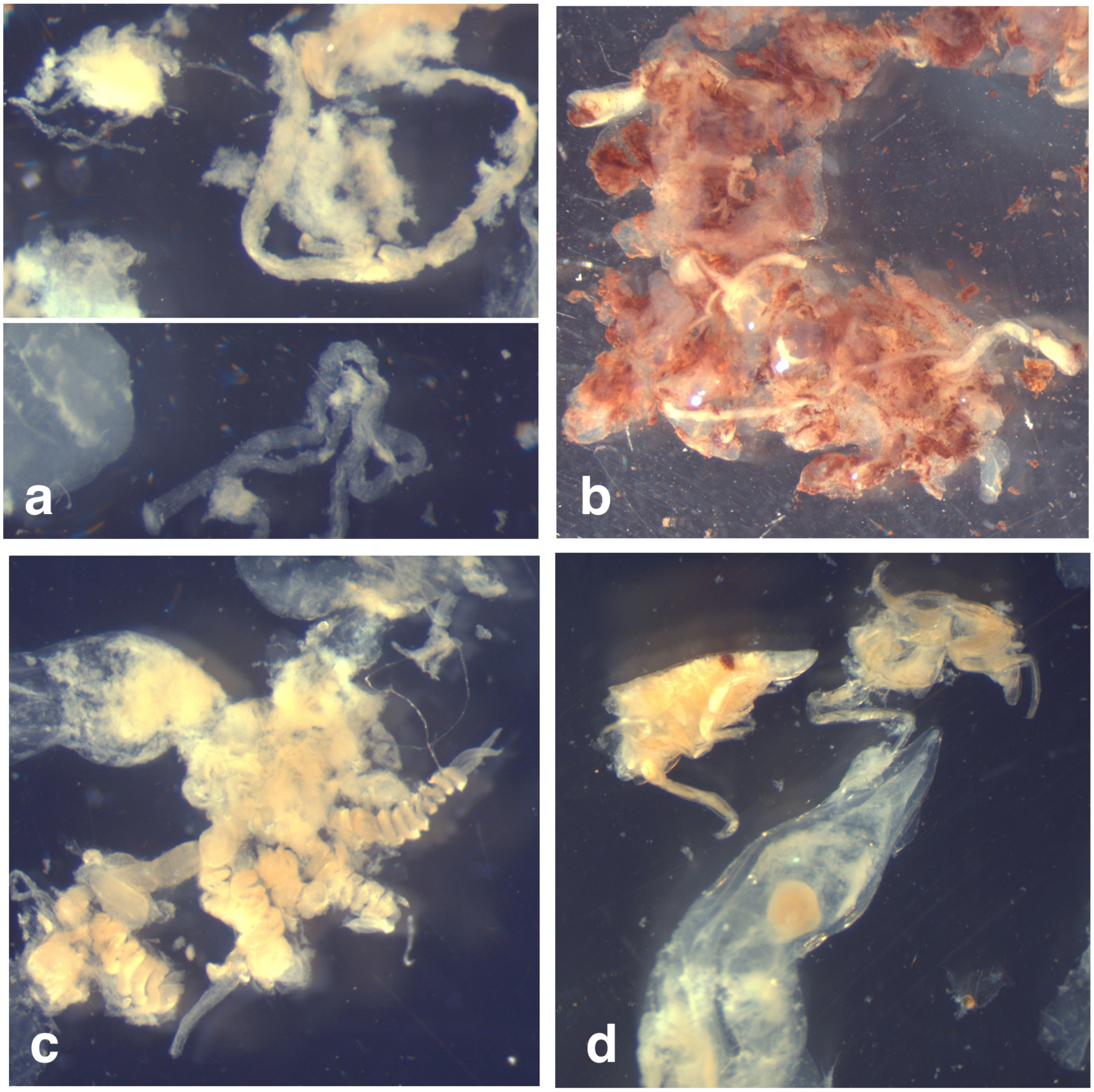

Figure S1: Gut contents of museum specimens of *Haliphron atlanticus.* (a) Tissue and tentacles resembling scyphozoan oral arms. (b) red pigmented tissue and tentacles from a coronate scyphozoan. (c) tentilla (coiled structures) and stem from a physonect siphonophore. (d) amphipod *Vibilia* (upper) and portion of a salp (middle, conical)
